# Assessment of Primary Colorectal Cancer CT Radiomics to Predict Metachronous Liver Metastasis

**DOI:** 10.3389/fonc.2022.861892

**Published:** 2022-02-28

**Authors:** Yue Li, Jing Gong, Xigang Shen, Menglei Li, Huan Zhang, Feng Feng, Tong Tong

**Affiliations:** ^1^Department of Radiology, Fudan University Shanghai Cancer Center, Shanghai, China; ^2^Department of Oncology, Shanghai Medical College, Fudan University, Shanghai, China; ^3^Department of Radiology, Affiliated Tumor Hospital of Nantong University, Nantong, China

**Keywords:** tomography, x-ray computed, colorectal neoplasms, neoplasm metastasis, liver neoplasms, machine learning

## Abstract

**Objectives:**

To establish and validate a machine learning-based CT radiomics model to predict metachronous liver metastasis (MLM) in patients with colorectal cancer.

**Methods:**

In total, 323 patients were retrospectively recruited from two independent institutions to develop and evaluate the CT radiomics model. Then, 1288 radiomics features were extracted to decode the imaging phenotypes of colorectal cancer on CT images. The optimal radiomics features were selected using a recursive feature elimination selector configured by a support vector machine. To reduce the bias caused by an unbalanced dataset, the synthetic minority oversampling technique was applied to resample the minority samples in the datasets. Then, both radiomics and clinical features were used to train the multilayer perceptron classifier to develop two classification models. Finally, a score-level fusion model was developed to further improve the model performance.

**Results:**

The area under the curve (AUC) was 0.78 ± 0.07 for the tumour feature model and 0.79 ± 0.08 for the clinical feature model. The fusion model achieved the best performance, with AUCs of 0.79 ± 0.08 and 0.72 ± 0.07 in the internal and external validation cohorts.

**Conclusions:**

Radiomics models based on baseline colorectal contrast-enhanced CT have high potential for MLM prediction. The fusion model combining radiomics and clinical features can provide valuable biomarkers to identify patients with a high risk of colorectal liver metastases.

## Introduction

Colorectal cancer (CRC) is the third leading type of cancer and the second most common cause of cancer death worldwide (1). Liver metastasis (LM) is the leading cause of death in patients with CRC (2). Approximately 50% of patients with CRC will develop LM over the course of their life, and surgical resection is the only treatment modality with curative intent and has 5-year and 10-year survival rates of 40% and 25% (3, 4). LM is a known prognostic predictor, and as a long-standing challenge in the treatment of CRC, the early identification of high-risk LM patients is crucial to improve clinical outcomes.

To the best of our knowledge, clinical parameters, including age, carcinoembryonic antigen (CEA) level, genetic mutations, and invasion of adjacent tissues [lymphovascular invasion (LVI) and perineural invasion (PNI)], are potential biomarkers to identify patients with a higher risk of distant metastasis (5–8). However, some predictors can only be obtained after radical resection and cannot be used as a guide for developing preoperative treatment strategies. The radiomics analysis method has the potential to noninvasively evaluate tumour heterogeneity objectively and quantitatively by analysing high-throughput information extracted from images (9). Evidence has gradually accumulated that computed tomography (CT) texture features are related to parameters such as tumour grade, tumour cellular processes and genetic mutations (10). In recent studies, some CT texture features have been linked to prognosis and clinical outcomes. Most of these features are based on the analysis of metastatic lesions, and few studies have focused on primary colorectal lesions. Effective and robust baseline biomarkers for the prediction of colorectal LMs are still lacking. The combination of radiomics and machine learning algorithms might unearth valuable features that can reflect the tumour heterogeneity of primary CRC and contribute to the prediction of the risk of metastasis.

The early identification of patients with a distinct likelihood of metachronous liver metastasis (MLM) may allow the consideration of different treatment strategies (e.g., neoadjuvant chemoradiotherapy) and a more intensive follow-up programme to improve the prognosis of patients. The purpose of this study was to determine whether the radiomics features of baseline colorectal contrast-enhanced CT can predict MLM in CRC patients.

## Materials and Methods

### Patients

This retrospective study was approved by the institutional review boards of all the participating institutions, and the requirement for informed consent was waived. We enrolled 323 CRC patients who underwent contrast-enhanced CT between October 2010 and January 2020. Dataset 1 (for model training, tuning, and internal validation) included patients enrolled from Fudan University Shanghai Cancer Centre, and dataset 2 (for independent external validation, n=75) included patients enrolled from Nantong Tumor Hospital. Dataset 1 was composed of a training cohort (n=171) and an internal validation cohort (n=77). The inclusion criteria were as follows: (1) histopathologically confirmed CRC; (2) performance of standard contrast-enhanced CT of the abdomen and pelvis before any treatment; (3) availability of clinical characteristics; and (4) availability of complete CT datasets. The exclusion criteria were as follows: (1) treatment (including radiotherapy or systemic chemotherapy) prior to initial CT examination; (2) LM before colorectal radical surgery; (3) presence of other tumour diseases during the same period; and (4) unavailable clinicopathologic or follow-up data.

### CT Scanning Protocol

All selected patients at both institutions underwent contrast-enhanced abdominal or pelvic CT with 64-row spiral CT scanners (Philips Healthcare, Siemens Healthcare) using a current of 200 mA and a tube voltage of 120 kV. All CT images were reconstructed with the standard reconstruction kernel, including 5.0 mm slice thickness, 5.0 mm increment, 1.4 or 0.9 pitch, 512×512 matrix and 4.11 cm field of view. The CT digital imaging and communications in medicine (DICOM) images were retrieved from the picture archiving and communication system (PACS).

### CT Radiomics Feature Model Development

**Figure 1:**
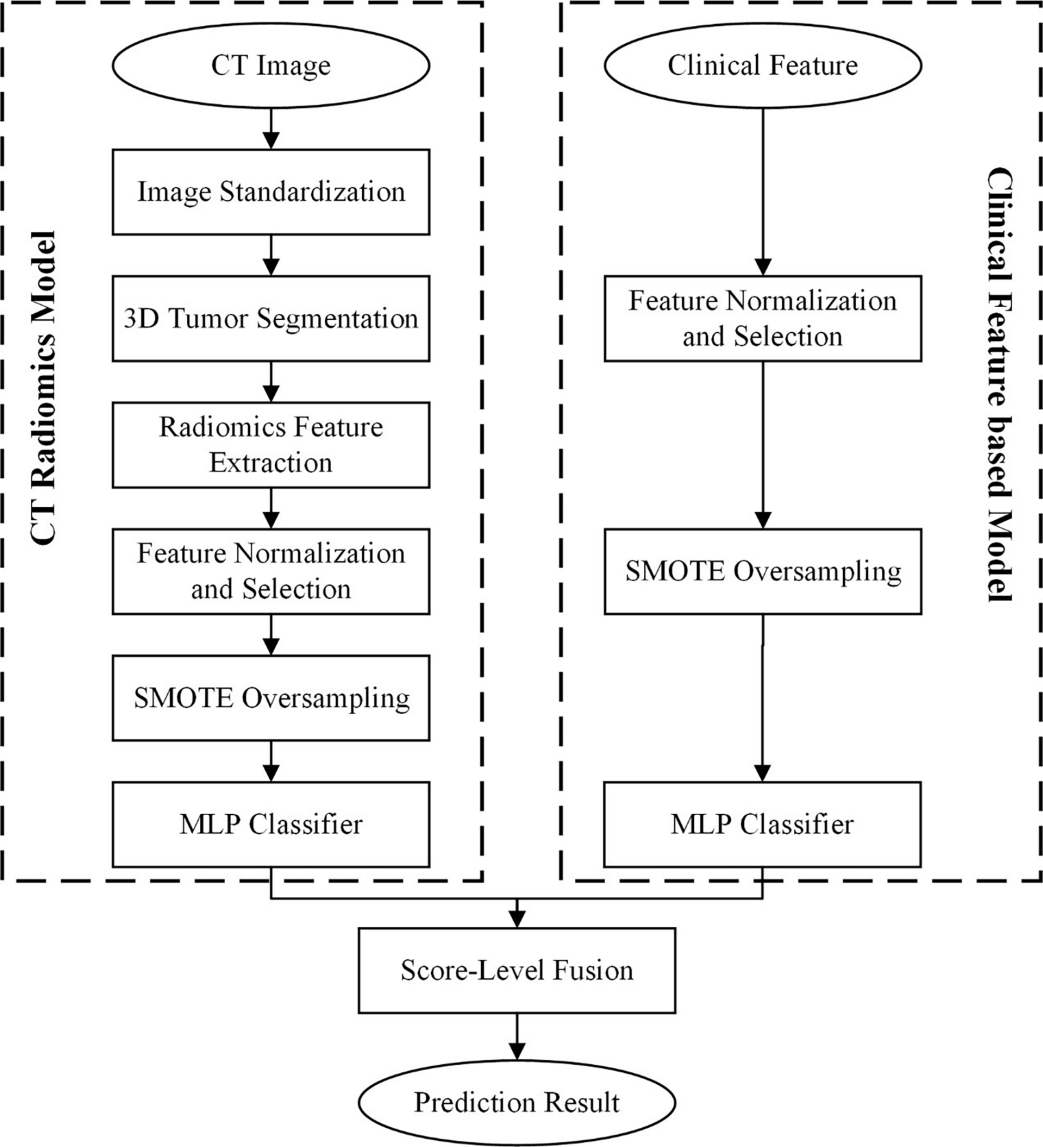
The flowchart of our proposed prediction model.

**Figure 1** illustrate the flowchart of our proposed prediction model. To evaluate the intra-class and inter-class agreement between different radiologists in segmentation process, we computed the Dice coefficient based on the segmentation results delineated by different radiologists. We initially chose 50 random colorectal contrast-enhanced CT images for ROI segmentation and feature extraction. The ROI segmentation was performed by two experienced radiologists independently. The Dice coefficients of inter-/intra-reader were higher than 0.85. To select the robustness of radiomics features, we calculated the inter-class/intra-class correlation coefficient (ICC) of each feature. As the ICC greater than 0.75 was considered good agreement, we just selected the radiomics features with inter- and inra-reader ICC values higher than 0.75. The boundaries of each tumour were delineated on CT images in a slice-by-slice fashion. ITK-SNAP software (version: 3.8.0, http://itksnap.org/) was used to segment the 3D tumour. **Figure S1** showed the diagram of our segmentation and workflow.

After segmenting the 3D tumour on the CT image, a cubic B-spline interpolation algorithm was applied to resample the CT scan with a new spacing of [1 mm, 1 mm, 5 mm]. Then, we computed the radiomics features by using Python programming software with the Pyradiomics package. A total of 1288 radiomics features were computed and extracted to decode the imaging phenotypes of 3D colorectal tumours. The original CT image and two types of transformed images, including wavelet images and Laplacian of Gaussian (LoG) images, were used to calculate image features. The LoG image filter was configured with δ values of 1, 2, 3, 4, and 5. Thus, the initial radiomics features consisted of 105 original image features, 728 wavelet image features and 455 LoG image features. The radiomics feature calculation progress following the IBSI (https://theibsi.github.io/) guidelines.

An L2-based normalization method was used to rescale each type of radiomics feature. Then, the recursive feature elimination (RFE) method was applied to reduce the dimensionality of feature spacing and remove the redundant image features. The linear support vector machine (SVM) classifier was selected as the estimator to configure the RFE feature selector. After selecting optimal radiomics features, an optimal imaging feature pool was selected from the initial radiomics features to build the classification model. Since our dataset was unbalanced, synthetic minority oversampling technique (SMOTE) was used to increase the number of minority samples in the dataset. In the CT radiomics development process, the SMOTE method was used to oversample only the training dataset. Finally, the multilayer perceptron (MLP) classifier was applied to build the classification model.

### Clinical Feature-Based Model Development

Clinical and pathological features were used to develop a clinical feature-based model to predict MLM in CRC patients. A min-max normalization scaler was first used to normalize the feature to a scale of [0,1]. Then, the optimal clinical features were selected according to the scores measured by using ANOVA F-value analysis. The minority samples were also resampled by using the SMOTE method. The MLP classifier was applied to build the classification model.

### CT Radiomics and Clinical Feature Fusion Model Development

To improve the model performance, a fusion model was developed by combining CT radiomics features and clinical features. A score-level fusion method was used to combine the prediction scores generated by the CT radiomics model and the clinical feature-based model. Three score fusion strategies, namely, the minimum score fusion strategy, maximum score fusion strategy, and weighted score fusion strategy, were used to build the CT radiomics and clinical feature fusion model. The prediction score of the minimum or maximum score fusion strategy was generated by comparing the prediction scores of each case yielded by the CT radiomics model and the clinical feature-based model to select the minimum or maximum score. The prediction score of the weighted score fusion strategy was generated by systematically increasing the weighting factor from 0.1 to 0.9 applied to the prediction scores generated by the CT radiomics model (or 0.9 to 0.1 applied to the prediction scores generated by the clinical feature-based model). A similar score-level fusion method was used in our previously reported study (11).

### Statistical Analysis

All data analyses were performed using Python 3.7.6. A P value of less than 0.05 was considered significant. A L2 based normalization method was used to rescale each type of the radiomics feature. The recursive feature elimination (RFE) method and linear support vector machine (SVM) classifier were implemented to select the optimal image features. And the optimal clinical features were selected according to the scores measured by using the ANOVA F-value analysis. Then, multi-layer perceptron (MLP) classifier was applied to build the classification model. The diagnostic performance of the models was evaluated by receiver operating characteristic (ROC) curves. The AUC of different models were compared using Delong test.

## Results

### Patient Characteristics

Based on the inclusion and exclusion criteria, a total of 323 patients were included, and 58.5% were male. The median age of the patients was 61 years (interquartile range, 53-69 years). Patients with MLM were defined as liver metastases that occurred after radical excision of the primary colorectal cancer (12). In dataset 1, 176 patients (71.0%) developed MLM. To avoid selection bias and reflect the natural distribution of morbidity, the patients in dataset 1 were divided into a training cohort (n=171) and a validation cohort (n=77) according to the date of the first visit. In dataset 2, 23 patients (30.7%) developed MLM. The baseline characteristics of the three cohorts are summarized in **Table 1**.

**Table 1:** Patient characteristics.

characteristic		Training cohort	Validation cohort 1	Validation cohort 2
		(n = 171)	(n = 77)	(n = 75)
Age SD [years]		57.7 ± 12.4	60.9 ± 12.6	64.9 ± 10.7
Gender (%)	Male	95 (55.6%)	46 (59.7%)	48 (64.0%)
	Female	76 (44.4%)	31 (40.3%)	27 (36.0%)
Location (%)	Right	71 (41.5%)	29 (37.7%)	25 (33.3%)
	Left	100 (58.5%)	48 (62.3%)	50 (66.7%)
MMR (%)	pMMR	122 (71.3%)	66 (85.7%)	68 (90.7%)
	dMMR	49 (28.7%)	11 (14.3%)	7 (9.3%)
KRAS (%)	WT	13 (7.6%)	5 (6.5%)	33 (44.0%)
	M	3 (1.8%)	4 (5.2%)	18 (24.0%)
	NA	155 (90.6%)	68 (88.3%)	24 (32.0%)
NRAS (%)	WT	16 (9.4%)	8 (10.4%)	0
	NA	155 (90.6%)	69 (89.6%)	75 (100.0%)
BRAF (%)	WT	16 (9.4%)	9 (11.7%)	17 (22.7%)
	M	0	0	31 (41.3%)
	NA	155 (90.6%)	68 (88.3%)	27 (36.0%)
Tumor stage (%)	T1	7 (4.1%)	2 (2.6%)	0
	T2	17 (9.9%)	11(14.3%)	5 (6.7%)
	T3	47 (27.5%)	24 (31.2%)	24 (32.0%)
	T4	100 (58.5%)	40 (51.9%)	46 (61.3%)
New tumour stage(%)	T1-2	24(14%)	13(13.9%)	5(6.7%)
	T3	47 (27.5%)	24 (31.2%)	24 (32.0%)
	T4	100 (58.5%)	40 (51.9%)	46 (61.3%)
Nodal stage (%)	N0	96 (56.1%)	40 (51.9%)	39 (52.0%)
	N1	53 (31.0%)	22 (28.6%)	27 (36.0%)
	N2	22 (12.9%)	15 (19.5%)	9 (12.0%)
Metastasis stage(%)	M0	161 (94.2%)	76 (98.7%)	75 (100%)
	M1	10 (5.8%)	1 ((1.3%)	0
Pre CA-19-9 (%)	Normal	150 (87.7%)	62 (80.5%)	68 (90.7%)
	Elevated	21 (12.3%)	15 (19.5%)	7 (9.3%)
Pre CEA (%)	Normal	98 (57.3%)	52 (67.5%)	43 (57.3%)
	Elevated	73 (42.7%)	25 (32.5%)	32 (42.7%)
LVI (%)	Positive	40 (23.4%)	62 (80.5%)	25 (33.3%)
	Negative	131 (76.6%)	15 (19.5%)	50 (66.7%)
PNI (%)	Positive	40 (23.4%%)	57 (74.0%)	15 (20.0%)
	Negative	131 (76.0%)	20 (26.0%)	60 (80.0%)

pMMR, proficient mismatch repair gene expressing; dMMR, deficient mismatch repair gene expressing; WT, wild type; M, mutant type; NA, not available; pre CA 19-9, the level of carbohydrate antigen 19-9 before any treatment; pre CEA, the level of carcinoembryonic antigen before any treatment; LVI, lymphatic vascular infiltration; PNI, peripheral nerve invasion.

### Intra-Observer and Inter-Observer Reproducibility of Radiomics Feature Extraction

The intra-observer ICC calculated based on two measurements of reader A was 0.983. The inter-observer ICC was 0.776. An ICC greater than 0.75 was considered good agreement. The results indicated stable intra and inter-observer feature extraction reproducibility.

### Selected Features for the Clinical Model

**Figure 2:**
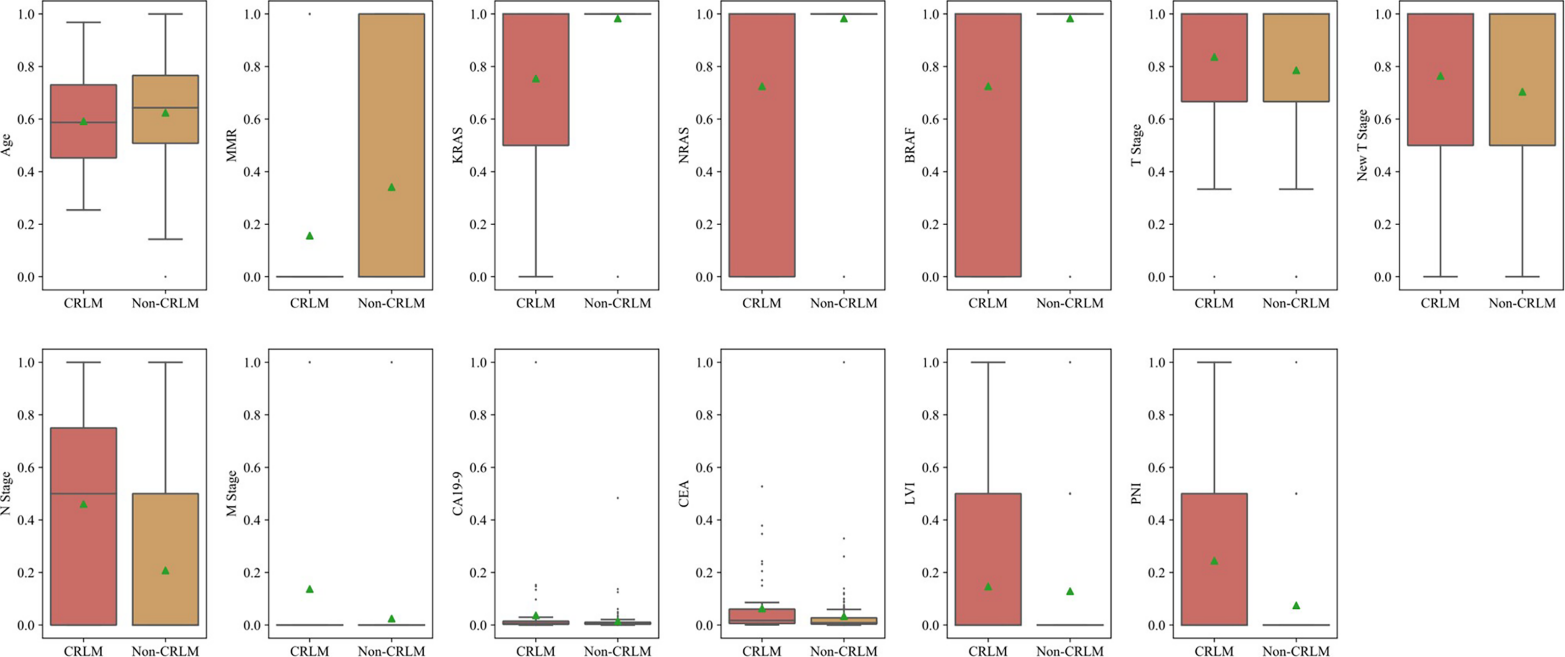
Box plot of clinical features of CRLM and Non-CRLM sets.

After normalization and ANOVA F-value analysis, clinical factors including age, mismatch repair (MMR) status, preoperative TNM stage, tumour markers (CEA and CA19-9), genetic mutations (KRAS, NRAS, and BRAF) and invasion of adjacent tissues (LVI and PNI) were significantly different between the CRC LM (CRLM) group and the non-CRLM group (P <0.05). The distributions of these features in the CRLM and non-CRLM groups are shown in **Figure 2**.

### Selected Features for the CT Radiomics Model

**Figure 3:**
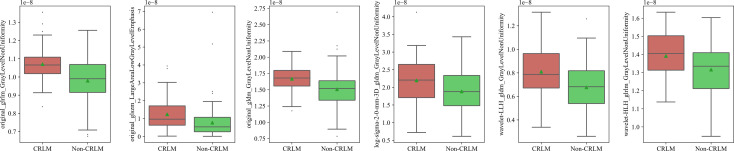
Box plot of radiomics features of CRLM and Non-CRLM sets.

In total, 1288 image features were selected by the SVM-RFE method. Six features passed the suggestive significance level (P <0.05), including three original image features, two wavelet image features and one LoG image feature. The distributions of radiomics features in the CRLM and non-CRLM groups are shown in **Figure 3**.

### Model Validation and Comparison

The radiomics feature-based model and clinical feature-based model had approximate performance in the validation sets [validation set 1, area under the curve (AUC): 0.70 ± 0.07 and 0.69 ± 0.08; validation set 2, AUC: 0.64 ± 0.07 and 0.68 ± 0.07]. Using score-level methods, combinations of various features of the radiomics and clinical models were adopted to validate which combination of features was more conducive to improving the prediction performance. No significant improvement in the prediction performance was found with the different weighted score fusion strategies (P<0.05). The minimum score fusion strategy did not contribute to the improvement of prediction performance. Ultimately, the fusion model using the maximum score fusion strategy achieved the best performance in validation set 1 (AUC: 0.79 ± 0.06, 95% CI 0.68-0.87, P<0.05) and validation set 2 (AUC: 0.72 ± 0.06, 95% CI 0.60-0.82, P<0.05). The detailed performance of different combinations of radiomics and clinical features from the respective models is provided in **Table 2**.

**Table 2:** The diagnostic performance of different combination of radiomics and clinical features.

Model	Validation Dataset 1		Validation Dataset 2	
	AUC	95% CI	AUC	95% CI
Rad	0.70 ± 0.07	[0.58, 0.80]	0.64 ± 0.07	[0.53, 0.74]
Cli	0.69 ± 0.08	[0.57, 0.82]	0.68 ± 0.07	[0.55, 0.80]
Minimum	0.68 ± 0.07	[0.56, 0.81]	0.70 ± 0.07	[0.58, 0.82]
Maximum	**0.79 ± 0.06**	**[0.68, 0.87]**	**0.72 ± 0.06**	**[0.60, 0.82]**
0.1*Rad+0.9*Cli	0.69 ± 0.08	[0.57, 0.82]	0.68 ± 0.07	[0.55, 0.80]
0.2*Rad+0.8*Cli	0.69 ± 0.08	[0.57, 0.82]	0.68 ± 0.07	[0.55, 0.80]
0.3*Rad+0.7*Cli	0.69 ± 0.08	[0.57, 0.82]	0.68 ± 0.07	[0.55, 0.80]
0.4*Rad+0.6*Cli	0.69 ± 0.08	[0.57, 0.82]	0.68 ± 0.07	[0.55, 0.80]
0.5*Rad+0.5*Cli	0.69 ± 0.08	[0.57, 0.82]	0.68 ± 0.07	[0.55, 0.80]
0.6*Rad+0.4*Cli	0.69 ± 0.08	[0.57, 0.82]	0.68 ± 0.07	[0.55, 0.80]
0.7*Rad+0.3*Cli	0.69 ± 0.08	[0.57, 0.82]	0.68 ± 0.07	[0.55, 0.80]
0.8*Rad+0.2*Cli	0.69 ± 0.08	[0.57, 0.82]	0.68 ± 0.07	[0.55, 0.80]
0.9*Rad+0.1*Cli	0.69 ± 0.08	[0.57, 0.82]	0.68 ± 0.07	[0.55, 0.80]

All data was rounded up to percentile. The prediction performance with the different weighted score fusion strategies differed in four decimal places and was found no significant improvement. The bold values refer to the diagnostic performance of our final fusion model.

Rad, Radiomics Feature based Model; Cli, Clinical Feature based Model.

The accuracy, sensitivity, specificity, positive predictive value, and negative predictive value of the internal and external validation sets for each model are reported in **Table 3**, which shows that the fusion model is the best among all models (P<0.05).

**Table 3:** The accuracy, sensitivity, specificity, positive predictive value, and negative predictive value of the internal and external validation sets for each model.

Model	Validation Dataset 1			Validation Dataset 2		
	Radiomics model	Clinical model	Fusion models	Radiomics model	Clinical model	Fusion models
Accuracy (%)	58.4	72.7	74.0	65.3	66.7	72.0
Sensitivity (%)	85.7	52.4	38.1	65.2	69.6	78.3
Specificity (%)	48.2	80.4	87.5	65.4	65.4	61.5
PPV (%)	38.3	50.0	53.3	45.5	47.1	47.4
NPV (%)	90.0	81.8	79.0	81.0	82.9	86.5
P Value	Validation Dataset 1			Validation Dataset 2		
Rad *vs* Fusion	4.8×10^-6^			3.6×10^-8^		
Cli *vs* Fusion	1.3×10^-7^			7×10^-5^		
Rad *vs* Cli	3.80			0.123		

PPV, positive predictive value; NPV, negative predictive value.

**Figure 4:**
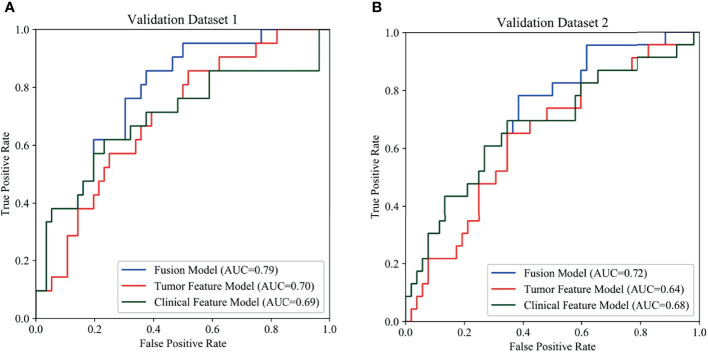
Comparison of prediction performance among the three models using different features. In the ROCs, the blue, red, green curves show the models based on fusion features, tumor features and clinical features, respectively. **(A)** ROCs of internal validation set, **(B)** ROCs of external validation set.

Overall, the tumour feature model (mean AUC: 0.78) performed similarly to the clinical feature model (mean AUC: 0.79), and the fusion model outperformed these models (mean AUC: 0.85). The receiver operating characteristic (ROC) curves of the three models are presented in **Figure 4**. The ROC curves of the three models in training set are presented in **Figure S2**.

## Discussion

This study aimed to develop and validate a machine learning-based CT radiomics model by using primary colorectal lesions to predict MLM. In this study, the fusion model that integrated radiomics features and clinical features significantly outperformed the radiomics and clinical models. It achieved good performance in both internal and external validations (AUC: 0.79, 95% CI 0.68-0.87; AUC: 0.72, 95% CI 0.60-0.82; P<0.05). Our results indicate that radiomics features have the potential to predict LMs and that the fusion model can provide valuable biomarkers to identify patients with a high risk of colorectal LMs.

The median survival of untreated patients with colorectal liver metastases is only 6.9 months, while median survival of patients undergoing radical resection of liver metastases is 35 months, with 5-year survival rates at around 40% (3, 13). Main clinical questions concern the ability of tools to accurately discern liver metastases and select patients for radical surgery. Our model could alert clinicians to patients with a higher risk of MLM. By implementing a more intensive follow-up programme or undergo neoadjuvant treatment for the high-risk MLM group, opportunities for radical resection of liver metastases can be offered and result in longer survival (14, 15).

Some clinical factors, including age, TNM stage, tumour markers (CEA and CA19-9), and genetic mutations (KRAS, NRAS, and BRAF), have been reported as risk factors for colorectal metastasis in previous studies (16–21). Although these factors have good predictive performance, they are only available after invasive operations. All of the above clinical features were also incorporated into our model. It should be noted that the new T stage mentioned in our clinical model combined stages T1 and T2 as one stage and compared it with stage T3 and stage T4. It showed predictive value for MLM in the study. The underlying mechanism has yet to be explored, and future validation in larger and more diverse samples is needed. In addition, this study included other factors, including MMR status and invasion of adjacent tissues (LVI and PNI). However, the AUC of clinical model were 0.69 ± 0.08 and 0.68 ± 0.07 in internal and external validation set, respectively. And the performance was not satisfactory.

By combining radiomics features including GrayLevelNonUniformity at GLRLM, GLDM, HLH, LoG filtration with sigma =2.0 and LALGLE at GLSZM, the predictive performance was improved to 0.79 and 0.72 in internal and external validation set, respectively.

There are no studies evaluating the performance of CT radiomics model based on the primary colorectal cancer to predict MLM. But several studies have investigated texture analysis of liver or rectal cancer to predict liver metastases. Beckers et al. reported texture analysis (uniformity at LoG filtration with sigma = 0.5) of liver could predict patients at risk of developing early LMs in ≤6 months but was not robust enough to identify patients at risk of developing metastases at later stages (22). In contrast, Lee et al. and Taghavi et al. showed that a CT radiomics nomogram based on the whole liver can provide a valuable assessment of the risk of MLM (23, 24). These findings suggest that radiomics and clinical features are complementary and mutually authenticated; thus, the comprehensive evaluation achieved a better prediction performance. Our study applied primary CRC features rather than liver features and found an increase in the predictive performance of the fusion model, which achieved good performance in the independent external validation. Some studies have shown that the heterogeneity of primary CRC tumours is linked to the aggressiveness of CRLMs and can predict the potential for LM (25–27). Based on similar inferences, Liu and Shu applied radiomics features of primary magnetic resonance imaging (MRI) rectal tumour images to predict synchronous LM (28, 29). Li et al. applied a single slice that included the largest tumour to predict MLM (30), and Liang et al. showed that radiomics models based on baseline rectal MRI had high potential for MLM prediction (31). Our study applied the assessment of whole primary lesions with CT scans rather than single slices to predict MLM and improved the model performance by using a machine learning algorithm.

Compared with previous studies, one strength of this study is the availability of an external test cohort. Independent external test cohorts contribute to evaluating the generalizability of predictive models (32). Another strength is the segmentation based on the entire 3D volume of the tumour and radiomics feature extraction using a machine learning algorithm, which can maximize the potential information underlying the images and thus identify the features with the highest predictive value. Moreover, this study focused on the primary lesions. Assessing the risk of LM using CT scans of primary CRC tends to be more routine, quick and economical, thus supporting its future application.

There are some limitations to our study. First, as a retrospective study with a limited dataset, selection bias and the presence of unknown confounders were possible. Although the patients were divided into a training cohort and a validation cohort according to the date of the first visit to avoid selection bias as much as possible, the small dataset may limit the generalization performance of the proposed model. Second, gene sequencing was not routinely applied, and thus, the genetic profiles were inadequate and formed a small sample. Third, the use of MLP classifier and maximum score strategy may be overoptimistic for the task. Further analysis will be made when additional cases are included in the advanced study. Finally, pre-treated patients were intentionally excluded to control for confounders and patients with other distant metastases were included. This may reduce the prediction efficiency of our model in general clinical practice. Prospective investigation using larger datasets and richer clinical profiles is needed to further validate the robustness and reproducibility of our conclusions. Despite these limitations, we hope that these findings will contribute to the prediction of MLM in patients with CRC.

In conclusion, combining CT radiomics and clinicopathologic features to develop a machine learning-based prediction model was feasible to predict MLM in patients with CRC. Before the proposed model is widely implemented in the clinic, more validation experiments need to be conducted by using diverse multicentre datasets, detailed genetic profiles and prospective studies.

## Data Availability Statement

The raw data supporting the conclusions of this article will be made available by the authors, without undue reservation.

## Author Contributions

YL and JG performed the statistical analysis. XS contributed to the image acquisition. ML and HZ performed the image segmentation. YL and JG wrote the draft of the manuscript. FF contributed to the external validation dataset. TT contributed to conception and design of the study. All authors contributed to manuscript revision, read, and approved the submitted version.

## Funding

This study has received funding from the National Natural Science Foundation of China (grant number: 81971687) and Natural Science Foundation of Shanghai (grant number: 20ZR1412700).

## Conflict of Interest

The authors declare that the research was conducted in the absence of any commercial or financial relationships that could be construed as a potential conflict of interest.

The reviewer FZ declared a shared affiliation, with no collaboration, with the authors to the handling editor at the time of the review.

## Publisher’s Note

All claims expressed in this article are solely those of the authors and do not necessarily represent those of their affiliated organizations, or those of the publisher, the editors and the reviewers. Any product that may be evaluated in this article, or claim that may be made by its manufacturer, is not guaranteed or endorsed by the publisher.

## Supplementary Material

Supplementary Figure S1 The diagram of study workflow.

Supplementary Figure S2 The diagnostic performances of three models on the training set.
