# Supplementary figures and images for: Assessment of Primary Colorectal Cancer CT Radiomics to Predict Metachronous Liver Metastasis
Source: Front Oncol. 2022 Feb 28;12:861892. doi: 10.3389/fonc.2022.861892 (PMC8919043; doi:10.3389/fonc.2022.861892)

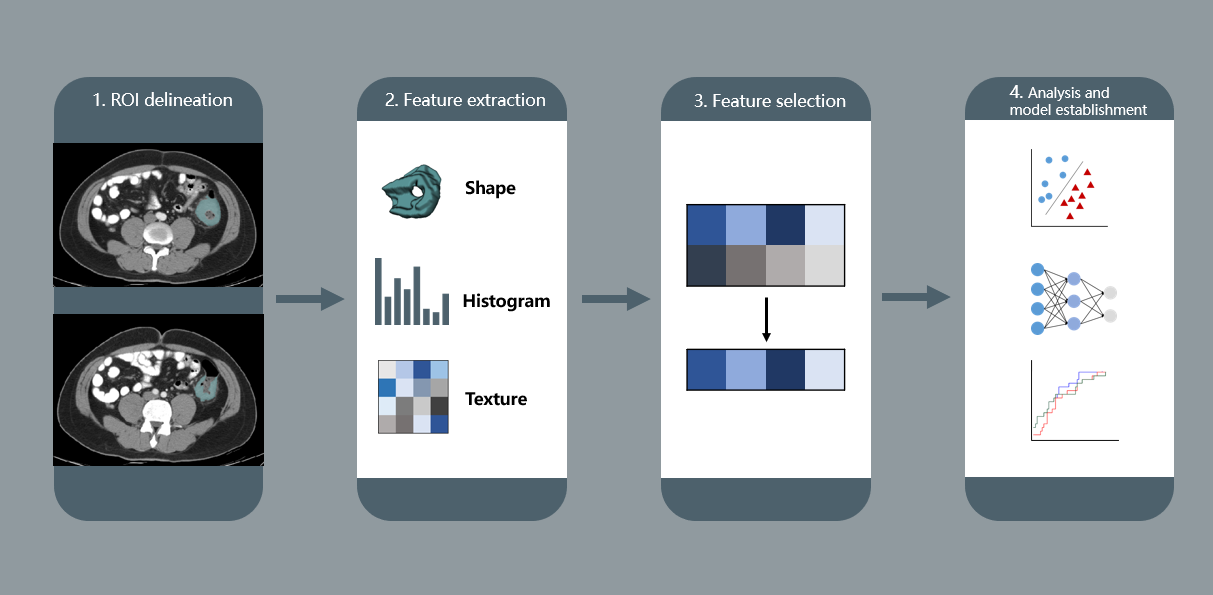

Supplement: Supplementary Figure S1 — The diagram of study workflow. [file Image_1.tif]

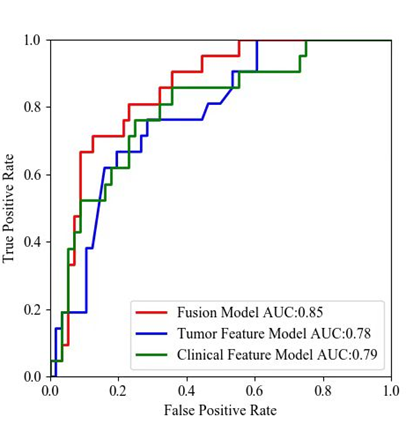

Supplement: Supplementary Figure S2 — The diagnostic performances of three models on the training set. [file Image_2.tif]
